# Supplementary material for: Reproductive Coercion by Intimate Partners: Prevalence and Correlates in Canadian Individuals with the Capacity to be Pregnant
Source: PLoS One. 2023 Aug 3;18(8):e0283240. doi: 10.1371/journal.pone.0283240 (PMC10399814; doi:10.1371/journal.pone.0283240)
Supplement: S1 Table — (DOCX) [file pone.0283240.s001.docx]

**S1 Table. Lifetime prevalence of items related to pregnancy coercion (N = 427).**

| Has an intimate partner… |  |
| --- | --- |
| threatened you so that you would become pregnant (e.g., threatening to leave you or to have a child with someone else)? | 2.8 % (12) |
| physically hurt you because you didn't want to get pregnant? | .7 % (3) |
| ever threatened to leave you or damage your reputation if you become pregnant? | 12.2 % (52) |
