# Supplementary material for: Reproductive Coercion by Intimate Partners: Prevalence and Correlates in Canadian Individuals with the Capacity to be Pregnant
Source: PLoS One. 2023 Aug 3;18(8):e0283240. doi: 10.1371/journal.pone.0283240 (PMC10399814; doi:10.1371/journal.pone.0283240)
Supplement: S2 Table — (DOCX) [file pone.0283240.s002.docx]

**S2 Table. Bloc 1 of the hierarchical logistic regression**

| Characteristics |  | Contraceptive sabotage | Pregnancy pressure | Pregnancy coercion |
| --- | --- | --- | --- | --- |
|  |  | Lifetime RC  OR (95% CI) | Lifetime RC  OR (95% CI) | Lifetime RC  OR (95% CI) |
| **Individual variables** |  |  |  |  |
| Age |  |  |  |  |
|  | 18 to 25 | 0.57 (0.28-1.17) | 0.42 (0.17-1.06) | 1.01 (0.25-4.03) |
|  | 26 to 35 | 0.67 (0.35-1.26) | **0.44 (0.20-0.97)*** | 0.89 (0.39-2.05) |
|  | 36 to 55 (Ref) |  |  |  |
| Sexual orientation |  |  |  |  |
|  | Bisexual | 1.74 (0.96-3.14) | 0.75 (0.32-1.73) | 0.76 (0.21-2.73) |
|  | Homosexual, Asexual, Pansexual, Questionning | 0.90 (0.53-1.53) | **0.39 (0.16-0.94)*** | 0.59 (0.21-1.68) |
|  | Heterosexual (Ref) |  |  |  |
| Economic perception |  |  |  |  |
|  | Insufficient or poverty | 1.88 (0.97-3.64) | **2.49 (1.16-5.36)*** | 0.72 (0.25-2.12) |
|  | At ease financially or Sufficient (Ref) |  |  |  |
| Education |  |  |  |  |
|  | High school; College | 1.30 (0.82-2.06) | **2.46 (1.29-4.70)**** | **3.25 (1.42-7.44)**** |
|  | University (Ref) |  |  |  |
| Occupation |  |  |  |  |
|  | Unemployed | 0.81 (0.32-2.05) | 1.61 (0.53-4.92) | 1.65 (0.47-5.72) |
|  | Student | 0.77 (0.47-1.26) | 1.05 (0.50-2.22) | 0.96 (0.33-2.79) |
|  | Worker (Ref) |  |  |  |
| Visible minority |  |  |  |  |
|  | Yes | 0.67 (0.31-1.45) | 1.61 (0.59-4.42) | 1.07 (0.25-4.56) |
|  | No (Ref) |  |  |  |
| Presence of a disability |  |  |  |  |
|  | Yes | **2.08 (1.02-4.23)*** | 0.74 (0.28-1.99) | 2.21 (0.70-6.95) |
|  | No (Ref) |  |  |  |

Note. Ref = reference category. *** = *p* < .001, ** = *p* < .01, * = *p* < .05. Contraceptive sabotage : χ^2^(10) = 19.90, *p* < .05; Cox & Snell *R^2^* = .05 Nagelkerke *R^2^* = .07. Pregnancy pressure : χ^2^ (10) = 24.68, *p* < .01; Cox & Snell *R^2^* = .06 Nagelkerke *R^2^* = .11. Pregnancy coercion : χ^2^ (10) = 11.42, *p* = n.s.; Cox & Snell *R^2^* = .06 Nagelkerke *R^2^* = .10
