# Supplementary material for: Reproductive Coercion by Intimate Partners: Prevalence and Correlates in Canadian Individuals with the Capacity to be Pregnant
Source: PLoS One. 2023 Aug 3;18(8):e0283240. doi: 10.1371/journal.pone.0283240 (PMC10399814; doi:10.1371/journal.pone.0283240)
Supplement: S3 Table — (DOCX) [file pone.0283240.s003.docx]

**S3 Table. Bloc 2 of the hierarchical logistic regression**

| Characteristics |  | Contraceptive sabotage | Pregnancy pressure | Pregnancy coercion |
| --- | --- | --- | --- | --- |
|  |  | Lifetime RC  OR (95% CI) | Lifetime RC  OR (95% CI) | Lifetime RC  OR (95% CI) |
| **Individual variables** |  |  |  |  |
| Age |  |  |  |  |
|  | 18 to 25 | 0.62 (0.30-1.30) | 0.49 (0.19-1.26) | 1.25 (0.30-5.26) |
|  | 26 to 35 | 0.78 (0.40-1.51) | 0.53 (0.23-1.18) | 1.00 (0.42-2.40) |
|  | 36 to 55 (Ref) |  |  |  |
| Sexual orientation |  |  |  |  |
|  | Bisexual | 1.53 (0.83-2.81) | 0.67 (0.28-1.56) | 0.69 (0.19-2.47) |
|  | Homosexual, Asexual, Pansexual, Questionning | 0.86 (0.50-1.49) | **0.39 (0.11-0.95)*** | 0.65 (0.22-1.93) |
|  | Heterosexual (Ref) |  |  |  |
| Economic perception |  |  |  |  |
|  | Insufficient or poverty | 1.87 (0.94-3.70) | **2.40 (1.11-5.19)*** | 0.64 (0.21-1.94) |
|  | At ease financially or Sufficient (Ref) |  |  |  |
| Education |  |  |  |  |
|  | High school; College | 1.26 (0.78-2.03) | **2.38 (1.24-4.54)**** | **2.80 (1.19-6.58)*** |
|  | University (Ref) |  |  |  |
| Occupation |  |  |  |  |
|  | Unemployed | 0.86 (0.33-2.24) | 1.78 (0.57-5.57) | 1.77 (0.48-6.58) |
|  | Student | 0.83 (0.50-1.39) | 1.13 (0.53-2.43) | 1.05 (0.35-3.20) |
|  | Worker (Ref) |  |  |  |
| Visible minority |  |  |  |  |
|  | Yes | 0.75 (0.33-1.70) | 1.74 (0.61-4.95) | 0.97 (0.22-4.41) |
|  | No (Ref) |  |  |  |
| Presence of a disability |  |  |  |  |
|  | Yes | 1.76 (0.85-3.64) | 0.66 (0.25-1.80) | 1.60 (0.50-5.11) |
|  | No (Ref) |  |  |  |
| **Relational variables** |  |  |  |  |
| Relational status |  |  |  |  |
|  | In a relationship, with a main partner or more than one partner | 0.63 (0.32-1.23) | 0.75 (0.32-1.77) | 0.44 (0.14-1.35) |
|  | Single, with no partner or a few partners (Ref) |  |  |  |
| Lifetime Intimate partner violence |  |  |  |  |
|  | Yes | **3.06 (1.89-4.95)***** | **2.99 (1.19-7.49)***** | 7.16 (.91-56.48) |
|  | No (Ref) |  |  |  |

Note. Ref = reference category. *** = *p* < .001, ** = *p* < .01, * = *p* < .05. Contraceptive sabotage : χ^2^(12) = 23.09, *p* < .001; Cox & Snell *R^2^* = .10 Nagelkerke *R^2^* = .14. Pregnancy pressure : χ^2^ (12) = 7.14, *p* < .05; Cox & Snell *R^2^* = .08 Nagelkerke *R^2^* = .14. Pregnancy coercion : χ^2^ (12) = 20.12, *p* = n.s.; Cox & Snell *R^2^* = .11 Nagelkerke *R^2^* = .17
