# Supplementary material for: Reproductive Coercion by Intimate Partners: Prevalence and Correlates in Canadian Individuals with the Capacity to be Pregnant
Source: PLoS One. 2023 Aug 3;18(8):e0283240. doi: 10.1371/journal.pone.0283240 (PMC10399814; doi:10.1371/journal.pone.0283240)
Supplement: S4 Table — (DOCX) [file pone.0283240.s004.docx]

**S4 Table. Bloc 3 of the hierarchical logistic regression (Final model)**

| Characteristics |  | Contraceptive sabotage | Pregnancy pressure | Pregnancy coercion |
| --- | --- | --- | --- | --- |
|  |  | Lifetime RC  OR (95% CI) | Lifetime RC  OR (95% CI) | Lifetime RC  OR (95% CI) |
| **Individual variables** |  |  |  |  |
| Age |  |  |  |  |
|  | 18 to 25 | 0.63 (0.30-1.32) | 0.49 (0.19-1.26) | 1.23 (0.29-5.28) |
|  | 26 to 35 | 0.83 (0.42-1.62) | 0.54 (0.24-1.21) | 1.21 (0.49-2.98) |
|  | 36 to 55 (Ref) |  |  |  |
| Sexual orientation |  |  |  |  |
|  | Bisexual | 1.48 (0.81-2.73) | 0.66 (0.28-1.55) | 0.57 (0.15-2.10) |
|  | Homosexual, Asexual, Pansexual, Questionning | 0.81 (0.47-1.42) | **0.39 (0.16-0.93)*** | 0.59 (0.19-1.79) |
|  | Heterosexual (Ref) |  |  |  |
| Economic perception |  |  |  |  |
|  | Insufficient or poverty | 1.74 (0.87-3.47) | **2.33 (1.07-5.08)*** | 0.53 (0.17-1.67) |
|  | At ease financially or Sufficient (Ref) |  |  |  |
| Education |  |  |  |  |
|  | High school; College | 1.27 (0.78-2.05) | **2.35 (1.23-4.49)*** | **2.70 (1.14-6.42)*** |
|  | University (Ref) |  |  |  |
| Occupation |  |  |  |  |
|  | Unemployed | 0.88 (0.34-2.30) | 1.83 (0.58-5.72) | 2.16 (0.57-8.21) |
|  | Student | 0.86 (0.51-1.44) | 1.15 (0.53-2.47) | 1.20 (0.38-3.73) |
|  | Worker (Ref) |  |  |  |
| Visible minority |  |  |  |  |
|  | Yes | 0.71 (0.31-1.62) | 1.70 (0.60-4.86) | 0.98 (0.21-4.53) |
|  | No (Ref) |  |  |  |
| Presence of a disability |  |  |  |  |
|  | Yes | 1.70 (0.82-3.52) | 0.66 (0.25-1.77) | 1.28 (0.39-4.18) |
|  | No (Ref) |  |  |  |
| **Relational variables** |  |  |  |  |
| Relational status |  |  |  |  |
|  | In a relationship, with a main partner or more than one partner | 0.66 (0.34-1.30) | 0.76 (0.32-1.79) | 0.45 (0.15-1.41) |
|  | Single, with no partner or a few partners (Ref) |  |  |  |
| Lifetime Intimate partner violence |  |  |  |  |
|  | Yes | **2.93 (1.80-4.76)**** | **2.94 (1.17-7.39)*** | 7.83 (.97-63.27)† |
|  | No (Ref) |  |  |  |
| **Community variables** |  |  |  |  |
| Social support |  |  |  |  |
|  | No | 1.46 (0.93-2.30) | 1.17 (0.61-2.24) | 2.44 (0.98-6.06) † |
|  | Yes (Ref) |  |  |  |

Note. Ref = reference category. *** = *p* < .001, ** = *p* < .01, * = *p* < .05, † = *p* = 0.054. Contraceptive sabotage : χ^2^(13) = 45.73, *p* < .001; Cox & Snell *R^2^* = .11 Nagelkerke *R^2^* = .15. Pregnancy pressure : χ^2^ (13) = 32.06, *p* < .01; Cox & Snell *R^2^* = .08 Nagelkerke *R^2^* = .14. Pregnancy coercion : χ^2^ (13) = 23.99, *p* < .05; Cox & Snell *R^2^* = .13 Nagelkerke *R^2^* = .20
